# MicroRNA miR-145-5p inhibits Phospholipase D 5 (PLD5) to downregulate cell proliferation and metastasis to mitigate prostate cancer

**DOI:** 10.1080/21655979.2021.1945361

**Published:** 2021-07-08

**Authors:** Juanni liu, Junhai Li, Yongtu Ma, Changbao Xu, Yigang Wang, Yanfeng He

**Affiliations:** a. Department of Oncology, NO.215 Hospital of shaanXi Nuclear Industry, Xianyang City, Shanxi Province, China; b. Department of Urology, NO.215 Hospital of shaanXi Nuclear Industry, Xianyang City, Shanxi Province, China; c. Department of Urology, The Second Affiliated of Zhengzhou University, Zhengzhou City, Henan Province, China; d. Clinical Pharmacology, NO.215 Hospital of shaanXi Nuclear Industry, Shanxi City, Shanxi Province, China

**Keywords:** MicroRNA, miR-145-5p, phospholipase d-5, pld-5, prostate cancer

## Abstract

Prostate cancer (PCa), a frequently detected malignant tumor, is the fifth leading global cancer mortality cause in men. Although research has improved the PCa survival rate, significantly reduced survival occurs among patients at the metastatic stage. MiRNAs, which are short non-coding proteins, are crucial for several biological roles, essential for PCa proliferation, differentiation, multiplication, and migration. The investigation aimed to explore miR-145-5p and PLD5 association and clarify their function in regulating proliferation in PCa cell lines.

The study used PC-3, LNCaP, DU-145 PCa, and RWPE-1 non-cancerous cell line, PCa, and BPH tissue specimens, and nude mice to validate results. MiR-145-5p and PLD5 manifestation were assessed through RT-qPCR. PLD5 and miR-145 binding was determined through dual-luciferase reporter gene assays. Validation of cell proliferation, migration, and invasion was assessed through MTT, scratch wound, and transwell assays, respectively.

The results indicated a downregulation of miR-145-5p level in PCa cell lines and tissues in comparison to the non-cancerous controls. PLD5 overexpression exerted a cancerous effect while mimicking of miR-145-5p reversed the PLD5-oncogenic effects and significantly inhibited PCa cells proliferation, migration, invasion, and metastasis.

In conclusion, the study revealed that miR-145-5p upregulated apoptosis and repressed migration, invasion, and metastasis of PCa via direct PLD5 modulation.

## Graphical Abtstract

**Figure:**
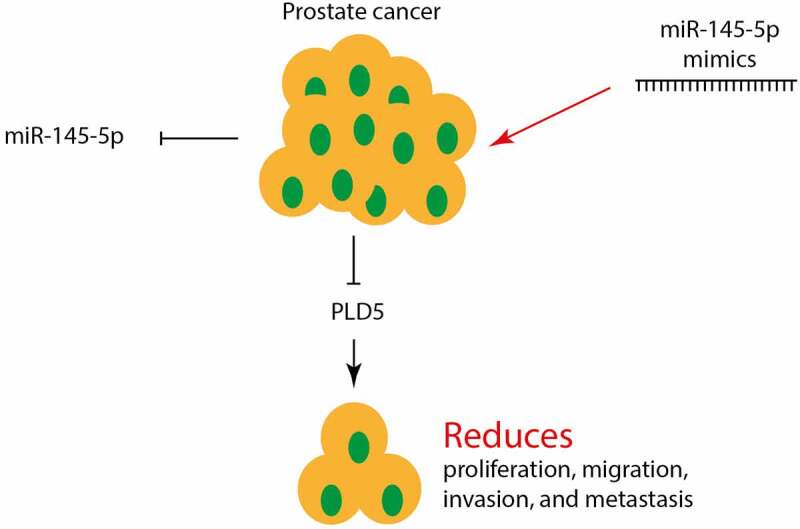

## 1. Introduction

Prostate cancer (PCa), the most frequently detected malignant tumor and the fifth leading global cancer mortality cause in men, with approximately 1.3 million new incidences, and 359,000 fatalities annually [1]. The current treatment methods for localized infection range from prostatectomy, active surveillance, or radiotherapy, mostly coupled with hormonal therapy. Although curative radiation approaches have led to prolonged lives, radiation resistance and subsequent clinical relapse are common in PCa [2]. Emphasis on molecular investigations preventing the occurrence of resistance can be essential for developing improved therapies.

Phospholipase D (PLD) is a transphosphatidylase enzyme essential in catalyzing the head group’s exchange on phosphodiester bonds that link various substrates. PLD uses water in hydrolyzing substrates of a phospholipid, for instance, phosphatidylcholine, to produce the phosphatidic acid (PA) membrane lipid, eventually discharging a solution of choline into the cytosol. Generally, six PLD isoforms exist in Humans [3]. PLD1 – PLD5 each function in encoding a pair of conserved catalytic aspartic acid, histidine, and lysine motifs that bend around itself, forming the entire catalytic site.

PLD and its PA generation are linked with various cellular effects such as migration, membrane fusion, growth, survival, and metabolism [4]. These functions contribute to numerous disease conditions, including neurodegeneration, inflammation, and cancer [5]. PLD upregulation occurs in various tumors, including renal, brain, breast, gastric, thyroid, colorectal, and expression correlated with various predictive measures [6].

MicroRNAs (miRNA) are small noncoding macromolecules, 18–24 nucleotides long, essential in regulating the post-transcriptional expression of mRNA [7]. MiRNAs function in several steps leading to neoplasm progression, including cell, proliferation, survival specialization, migration, or apoptosis. Consequently, miRNAs activities eventually lead to cancer’s pathogenesis, thus are regarded as gene regulator family [8]. Their dysregulation is demonstrated in tumor cell lines, clinical samples or xenografts. The occurrence of practically half of the well-defined miRNAs in the genome fragile zones hints at their important role in the progression of cancer [9].

So far, several miRNAs have been reported to play regulatory role in the progression, regression, invasion and metastasis of the PCa [10]. For instance, miRNA-222-3p served regulatory role in metastatic PCa by negatively regulating SNAP91 expression [11]. Moreover, overexpression of miR-181a-5p inhibited the expression of PTEN and regulated the PI3K/AKT/mTOR signaling pathway [X. 12]. It was studied that targeting of MTDH by miR-145-5p or miR-145-3p is associated with PCa prognosis, thereby regulating the growth and metastasis of PCa cells [D. 13]. Meanwhile, it was also reported that miR-145-5p negatively regulates the level of SOX2 which exerts crucial role in PCa tumorigenesis and proliferation [2]. However, to completely understand the exact role of miR-145-5p in PCa, further studies are required. Therefore, we designed this current investigation and hypothesized the association of miR-145-5p in the PCa suppression via PLD5 targeting. In addition, the study aimed at determining the impacts of miR-145 expression/overexpression on PCa, determining whether PLD5 is the target of miR-145-5p in PCa, and finally, to clarify the PLD5 knockdown effect in PCa. We noted the essential miR-145-5p role in growth, invasion, and migration arrest of PCa cells via the inhibition of PLD5.

## 2. Materials and methods

### 2.1 Human samples

We collected 20 prostate tumor samples (PCa) from 20 individuals (age = 65.2 ± 12) and 18 tissue samples from individuals with BPH (benign prostatic hyperplasia; age = 67.4 ± 11) after obtaining informed consent at our hospital between March 2016 and September 2019. Characteristics of human specimens are provided in Table 1. All the specimens were instantaneously frozen in liquid nitrogen and kept at −150°C for future use.

**Table 1.** Characteristics of human samples

Parameter	Prostate cancer	BPH
n	18	18
Age at diagnosis; Median, (Range)	71 (57–89)	71 (52–78)
Follow-up months; Median, (Range)	53 (0–169)	44 (4–57)
PSA (ng/ml); Median, (Range)	28.7 (2–276.4)	5.9 (0.6–36)
Gleason Score (n = 18)		
G6, n (%)	1 (5%)	-
G7, n (%)	5 (27%)	-
G8-10, n (%)	12 (66%)	-
Clinical T stage (n = 18)		
Tx, n (%)	1 (5%)	N/A
Tx, n (%)	7 (39%)	N/A
Tx, n (%)	5 (27%)	N/A
Tx, n (%)	3 (16%)	N/A
Tx, n (%)	2 (11%)	N/A

### 2.2 Cell culture and mRNAs transfection

PC3, LNCaP, DU145, and PCa cell lines were used for miR-145-5p functional analysis. Immortalized non-cancerous RWPE-1 cell lines were used as control. All the cell lines were obtained from the Chinese Academy of Science cell Bank and grown using DMEM (Dulbecco’s Modified Eagle’s medium) medium (Gibco-BRL, Bethesda, MA), supplemented with 10% fetal bovine serum (FBS) at 37°C in a wet and 5% CO_2_ incubator. Media change was done every 48 hrs [X. 14].

### 2.3 Cell transfection

PC3 and LNCaP experimental cells were divided into different groups then transfected using miR-145-5p mimics, PLD5-siRNA, PLD5-OE, and the comparable controls (miR-NC, si-NC, and pcDNA), respectively. Oligonucleotides and plasmids were synthesized using a Dharmacon kit. Each group contained approximately 3 × 10^5^ cells. The cells were transiently transfected using Lipofectamine 2000^TM^ (Invitrogen, Carlsbad, CA, USA). Summarily, cells (within approximately 70% confluency) grown in 6-well plates and transfected with the required oligonucleotides and plasmids [15].

### 2.4 Cell viability

Using 3-(4,5-Dimethylthiazol-2-yl)-2,5-diphenyl tetrazolium bromide (MTT) (Solarbio Life Sciences Co., Beijing, China), cell viability assay was conducted. Approximately, 1 × 10^4^ cells/well were incubated in 96-well plates and grown at 37°C for numerous time intervals. The cells were later washed, and 20 µl MTT reagent was supplemented to every well. The cells were grown further for 4 h. Afterward, DMSO (150 µl) was added after the removal of MTT. Finally, optical cell density (OD) was determined in a microplate reader at 562 nm [16].

### 2.5 RT-qPCR

Isolation of total RNA was done using TRIGene reagent as per the manufacturer’s guidelines. Processing of cDNAs was done through RNA reverse transcription using the GoScriptTM Reverse Transcription kit (Promega). qPCR was performed via SYBR Fast qPCR Mix for PLD5 and has-miR-145-5p. The primer sequences used were as follows has-miR-145-5p F-GTCCAGTTTTCCCAGGAATCCCT and R-TGGTGTCGTGGAGTCG; PLD5: F – GGAAAATATTCCTGAAGGCCTT and R – GTAAGCGGTCATGTTCATGTAC; Actin: F-GCACCACACCTTCTACAATG and R – TGCTTGCTGATCCACATCTG was used as the control. Samples were then run under various cycling parameters as follows: 95°C for 5 min, 95°C for 30 sex, then 35 cycles at 60°C for 30 sec and 72°C for 30 sec. After the amplification of PCR, comparative quantification of miR-145-5p and PLD5 was calculated via the 2^−ΔΔCq^ method [17].

### 2.6 Cell scratch wound assay

Tumor cells were plated and grown for 24 h at 37°C. A scratch was centrally introduced into the plate with a sterile pipette tip. Later, the wells were carefully washed thrice in PBS then a serum-free medium was added. Migration of cells was observed in an inverted microscope per 24 h [15].

### 2.7 Cell invasion experiment

To determine cell invasion, eight pore inserts pre-coated with Matrigel were used. After transfecting the cells for 48 h, the cells were obtained and re-suspended in a serum-free DMEM medium. A suspension of approximately 1 × 10^5^ cells in every mL was then cultured in the upper chambers. The lower chamber was filled with a complete DMEM medium containing 10% FBS. Invasion of cells was observed 24 hours later. Non-invading cells in the upper chamber were then removed using a swab of cotton wool. Cells that eventually settled on the lower membrane were exposed to fixation using 4% paraformaldehyde and subsequent staining in 0.05% crystal violet. Microscopic observations were randomly made of at least four fields, and cells were finally quantified using NIH-ImageJ software [X. 14].

### 2.8 In vivo *experimental mouse model*

Mice were also utilized in the investigation and were duly sustained following the recommendations stipulated by our Institute on Laboratory Animals Use. Nude mice (8-week-old males) were purchased from Hanghai SLAC Laboratory Animals Co., Ltd. (Shanghai, China), and housed in a specific pathogen-free animal facility. PC3 cells were resuspended in 100 μL serum-free medium, mixed with 40% Matrigel (BD Biosciences) and injected (2 × 106/site) subcutaneously into hind flank of each mouse (n = 20). When the average tumor volumes reached 70 mm3, tumor bearing mice were randomly divided into two groups, control and experimental. Then, 10 µg of mimic miR-145-5p or control oligo complexed with Lipofectamine 3000 (Invitrogen) in 50 µL Opti-MEM was injected intratumorally at an interval of 3 days a total of seven times. All mice were sacrificed 5 weeks post PC3 cell implantation. After sacrificing, the tumor weight was calculated [G. M. 18].

### 2.9 Dual-Luciferase assay

MiR-145-5p transcribed sequence or empty vector and luciferase reporter gene consisting of 3ʹ-UTR of PLD5 mutant or wild-type fragment were co-transfected into 293 T cells (previously grown in a 96-well plate) using Lipofectamine 2000. Cells were mowed 2 days subsequent transfection, and used dual-luciferase reporter system to access luciferase activity [17].

### 2.10 Western blot analysis

Cells protein extraction was done by RIPA lysis solution and quantified using BCA kit, then the protein aliquots separation was done by SDS-PAGE gel. The subsequent proteins were relocated to PVDF membranes, blocked with 5% milk and incubated with anti-PLD5, – snail, – cadherin, vimentin and Akt primary antibodies (1:1,000) at 4°C overnight. Antibody against actin was used as the internal housekeeping control. Incubation of the membrane with the appropriate HRP-conjugated secondary antibodies (1:10,000) was then done. The bands were finally pictured using Immobilon^TM^ HRP substrate (Millipore). Β-Actin was used as internal control [19].

### 2.11 Statistical analysis

Data analysis was done using SPSS 21.0. The data was measured as Mean ± SD. Comparison of the experimental sets difference was carried out using one-way ANOVA or Student’s t-test. The *P < 0.05 and **P < 0.01 were set as significant values.

## 3. Results

### 3.1 Expression of miR-145-5p is reduced during PCa

**Figure 1.**
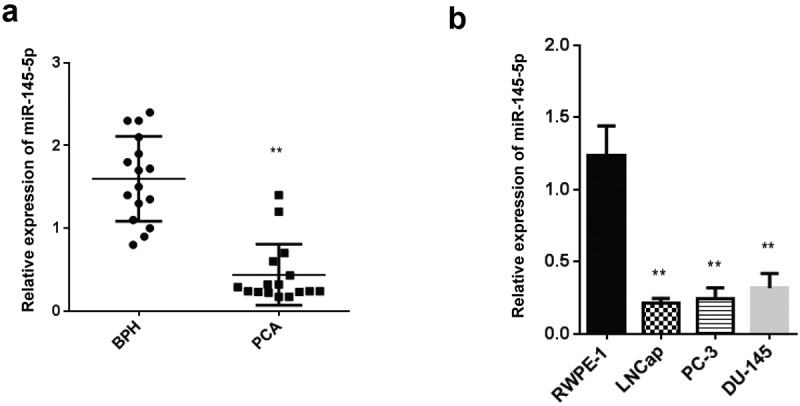
Level of miR-145-5p is downregulated in PCa

It has been previously reported that miR-145-5p play regulatory role in the PCs. However, to completely understand the exact role of miR-145-5p in PCa, further studies are required. Therefore, in this study, we aimed to determine the expression miR-145-5p in PCa, RT-qPCR. Our findings reported a significantly reduced miR-145-5p level in PCa tumors compared to the BPH tissues (Figure 1a). Similarly, we carried out RT-qPCR to determine the expression of miR-145-5p in LNCap, PC-3 and DU-145 PCa cells. The outcomes showed significantly reduced miR-145 in LNCap, PC-3 and DU-145 lines than RWPE −1 normal cells (Figure 1b).

A-B: Level of miR-145-5p in PCa tissue (A) and PCa cell lines (B). *P < 0.05 and **P < 0.01.

### 3.2 *The miR-145-5p mimics inhibits the growth and invasion of Prostate Cancer* in vitro

**Figure 2.**
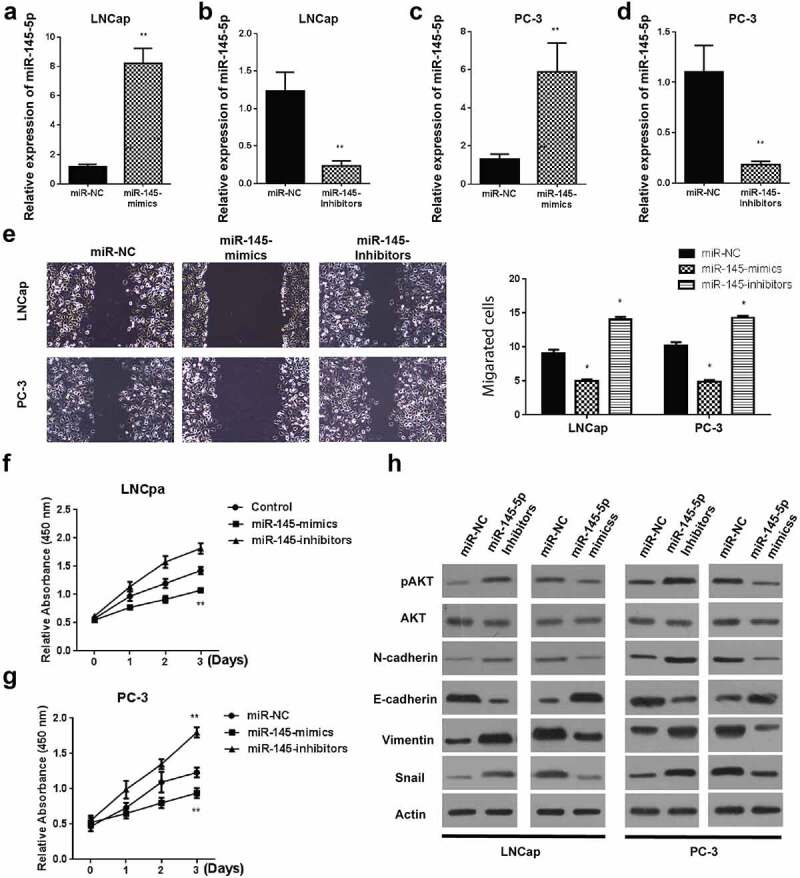
Modulating miR-145-5p regulates invasion and metastasis in PCa *in vitro.*

Transfection of miR-145-5p mimics in Prostate cancer cell line confirmed a significant increase in miR-145-5p expression in PCa LNCaP cells, as demonstrated in Figure 2a. However, the expression of miR-145-5p was considerably suppressed following the LNCaP cell lines treatment with miR-145-5p inhibitors compared to the control, as demonstrated in Figure 2b. Similarly, miR-145-5p mimics assessment in PC-3 cell line confirmed a significant miR-145 expression elevation than the control, as demonstrated in Figure 2c, while miR-145-5p expression was significantly reduced following the treatment of PC-3 cells with miR-145-5p inhibitors as compared to the control, as demonstrated in Figure 2d.

A-D: Relative expression level of miR-145-5p in LNCap cells post mimics transfection (A) and Inhibitors transfection (B). Relative expression of miR-145-5p in PC-3 cells after transfection with mimics (C) and Inhibitors (D); E: Cell migration in LNCap and PC-3 cells after transfection with miR-145-5p mimics and inhibitor; F-G: cell growth in LNCap (F) and PC-3 (G) cells after transfection with miR-145-5p mimics and inhibitor; H: western blotting for proteins involved in growth, metastasis and invasion of PC cells. *P < 0.05 and **P < 0.01.

Cell migration was then determined using the scratch wound assay. Experimental assessment 24 hours after creating scratch wounds demonstrated significantly reduced cell migrations in LNCaP and PC-3 cells (Figure 2e) in miR-145-5p mimics than the miR-145-5p inhibitors and negative control cells, respectively.

Further, viability assessment of cell viability was done using the MTT assay. Our observation demonstrated a significant reduction in viable LNCaP cells (figure 2f) and PC-3 cells (Figure 2g), following the LNCaP and PC-3 cells’ transfection with miR-145-5p mimics than the transfection with miR-145-5p inhibitors or empty vectors, respectively.

Finally, cell proliferation and metastasis capabilities were investigated by assessing proteins’ expression following treatment of prostate tumor cells with miR-145-5p mimics or inhibitors through western blotting. The outcomes indicated a significant reduction in N-cadherin, E-cadherin, Vimentin, and snail protein expression in both LNCaP and PC-3 cells incubated with miR-145-5p mimics compared to controls (Figure 6h). Expression of p-AKT protein was also significantly reduced in both the cell lines following treatment with miR-145-5p mimics compared to the controls, as demonstrated in Figure 6h. However, we observed contrasting results in cells incubated with miR-145-5p inhibitors. These results showed that suppressive effects of miR-145-5p in prostate cancer.

### 3.3 Mimicking miR-145-5p reduced PCa tumor size and tumor weight

**Figure 3.**
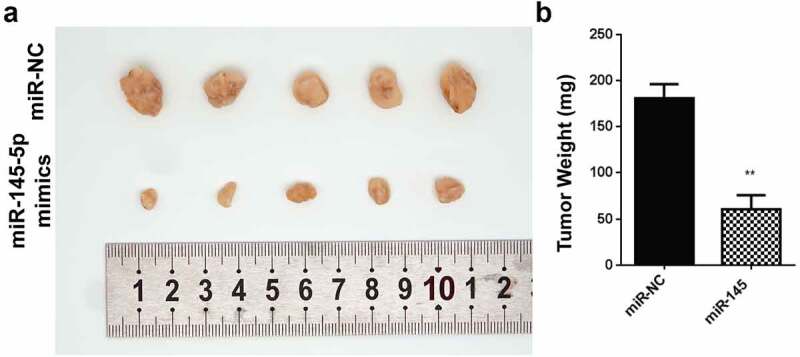
Overexpression of miR-145-5p reduces the tumor growth

The in vitro data prompted our investigation of roles played by miR-145-5p in the development of the tumor. The system was then assayed for its tumor formation ability For in vivo, we constructed and subcutaneously injected cells transfected with lentivirus carrying miR-145-5p in a small number of mice (n = 5). Averagely, the tumor volume was significantly reduced in the experimental mice that had an injection with cells expressing miR-145 mimics, compared to with empty vector, Figure 3a. The tumor weight in mice harboring cells expressing miR-145-5p mimics injection was significantly reduced compared to cells injected with an empty vector (Figure 3b).

A-B: Tumor size (A) and Tumor weight (B) after delivery of cells transfected with miR-145-5p overexpression vector. *P < 0.05 and **P < 0.01.

### 3.4 PLD5 is a target protein of miR-145 in prostate cancer

**Figure 4.**
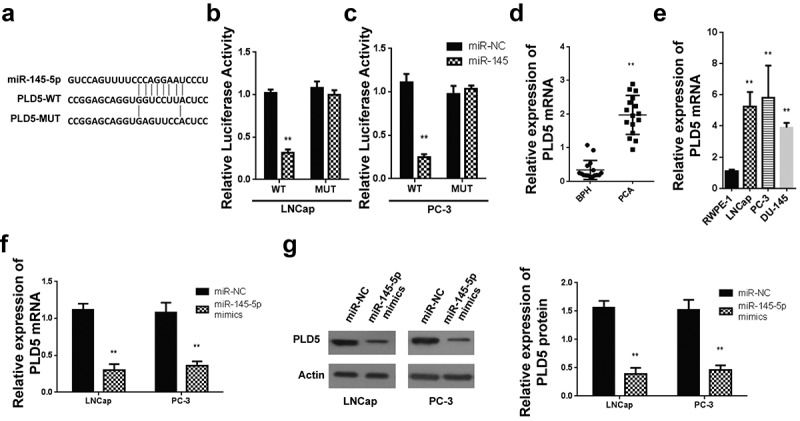
Targeting of PLD5 by miR-145-5p in PC cells

Assessment of miR-145-5p target gene by a miRNA prediction tool (targetscan) reported that PLD5 holds a site it uses for binding miR-145-5p in the 3ʹUTR region shown, as demonstrated in Figure 4a. For confirmation, a dual-Luciferase reporter assay was used for the detection of luciferase activity. The miR-145-5p, mutant, and wild-type PLD5 3ʹUTR was shown in Figure 4a. PLD5 wt or Mut 3′-UTR in LNCaP-luciferase analysis in LNCaP – cells post-transfected using miR-145-5p mimics showed a significantly decreased activity of luciferase in LNCap cells transfected with WT PLD5 and miR-145-5p mimics (Figure 4b). Nevertheless, miR-145-5p did not significantly change relative luciferase activity following the transfection of cells using the MUT PLD5 plasmid.

A: 3′UTR region of PLD5 containing the binding site for miR-145-5p in WT and MUT plasmids; B-C Luciferase activity of PLD5 WT or MUT 3′-UTR with miR-145-5p mimics or NC in LNCap (B) and PC-3 (C) cells; D-E: Expression of PLD5 mRNA in PCa specimens (D) and PCa cell lines (E); F-G Expression of PLD5 mRNA (F) and protein (G) in PCa cell lines after miR-145-5p mimics transfection. *P < 0.05 and **P < 0.01.

Similarly, PLD5 wt or Mut 3′-UTR in PC-3 luciferase analysis in PC-3 cells post-transfected using miR-145-5p mimics indicated a considerably inhibited luciferase activity in PC-3 cells with WT PLD5 and miR-145-5p mimics, as shown in Figure 4c. Contrarily, miR-145-5p did not significantly change relative luciferase activity following the transfection of the cells using the MUT PLD5 plasmid.

Later, RT-qPCR was used in evaluating the PLD5 mRNA expression in PCa tissues, and PC cell lines. According to the findings, PLD5 mRNA expression was significantly increased in PCa tissues than in the BPH control tissue (Figure 4d). Similarly, relative expression of PLD5 was increased in PC-3, DU-145 and LNCaP compared to the RWPE-1 cells (Figure 4e).

Further PLD5 mRNA expression analysis was done for LNCaP and PC-3 cells transfected using miR-145-5p mimics-NC, miR-145-5p-PLD5 mimics, inhibitors-NC, and miR-145-5p inhibitors. According to the findings, PLD5 mRNA levels were significantly repressed following miR-145-5p mimics transfection in both LNCaP (figure 4f) and PC-3 cells (figure 4f) paralleled to miR-NC mimic. Eventually, a western blotting assay was used to study PLD5 protein expression in LNCaP, and PC-3 miR-145-5p mimics transfected cells. Our observations indicated significant downregulation of PLD5 proteins in LNCaP (Figure 4g) and PC-3 cells (Figure 4g) transfected using miR-145-5p mimics, but not in the cells transfected using miR-NC. According to these data, miR-145-5p binds to the PLD5 3ʹUTR and controls mRNA and protein levels.

### 3.5 PLD5 silencing represses PCa cell growth, invasion and metastasis

**Figure 5.**
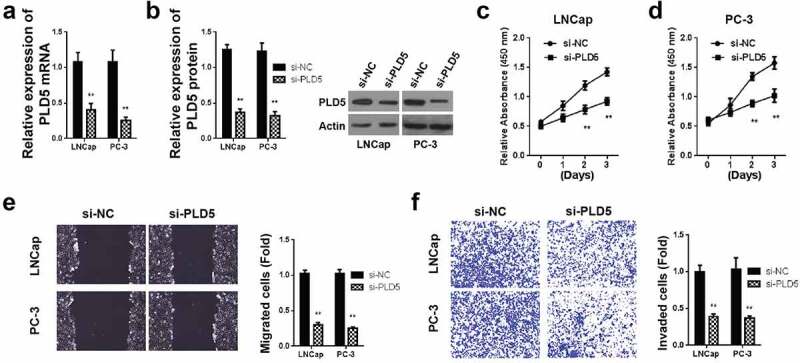
Knockdown of PLD5 suppresses PCa invasion and metastasis

To study the effect of PLD5 on prostate cancer, LNCaP, and PC-3 cells were transfected using various PLD5 siRNAs, and then the expression of PLD5 protein was assessed using western blotting. As shown in Figure 5a, the PLD5 mRNA expression was reduced in LNCaP cells transfected with si-PLD5. The western blotting analysis also showed a significantly reduced PLD5 expression in LNCaP cells transfected with si-PLD5, as shown in Figure 5b. Similarly, the PLD5 mRNA expression decreased in PC-3 cells transfected with si-PLD5 (Figure 5a). The western blotting analysis also showed a significantly reduced PLD5 expression in PC-3 cells transfected with si-PLD5, as shown in Figure 5b.

A-B: Expression of PLD5 mRNA (A) and protein (B) in PCa cell lines after si-PLD5 transfection. C-D: Cell growth in LNCap (C) and PC-3 (D) cells after transfection with si-PLD5; E: Cell migration in LNCap and PC-3 cells after transfection with si-PLD5; F: Invaded cells in LNCap and PC-3 cells after transfection with si-PLD5. *P < 0.05 and **P < 0.01.

We then evaluated cell proliferation by MTT assay in LNCaP and PC-3 cells post-transfected with si-PLD5 or si-NC. According to the results, the proliferation activities were significantly reduced after transfection with si-PLD5 in both LNCaP and PC-3 cells, respectively (Figure 5c and Figure 5d). Cell migration was also determined in LNCaP and PC-3 cells post-transfected with si-PLD5 or si-NC. Experimental outcome assessment 24 hours post-scratch wounds creation demonstrated a significant reduction of cell migrations in LNCaP and PC-3 cells post-transfected with si-PLD5 (Figure 5e) negative control cells, respectively.

Finally, cell colony formation analysis was done in LNCaP and PC-3 cells post-transfected with si-PLD5 or si-NC. Experimental outcome assessment 24 hours post-transfection demonstrated a significant reduction of cell colonies in LNCaP and PC-3 cells post-transfected with si-PLD5 (figure 5f) negative control cells, respectively. According to these data, PLD5 silencing negatively affects the migration and growth of prostate cancer.

### 3.6 Transfection of miR-145-5p ameliorates cell proliferation and metastasis of prostate cancer

**Figure 6.**
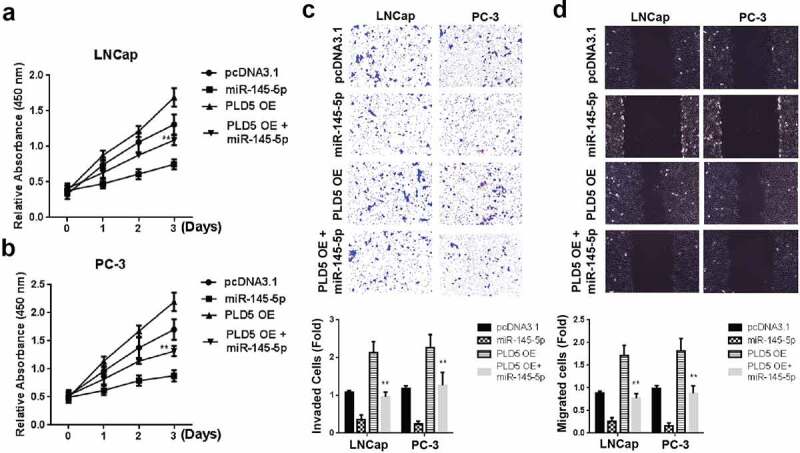
Mimicking miR-145-5p rescues the PC tumor growth, invasion and metastasis

A-B: Cell growth in LNCap (A) and PC-3 (B) cells after transfection with miR-145-5p mimics and PLD5 OE plasmid; C: Invaded cells in LNCap and PC-3 cells after transfection with miR-145-5p mimics and PLD5 OE plasmid; D: Cell migration in LNCap and PC-3 cells after transfection with miR-145-5p mimics and PLD5 OE plasmid. *P < 0.05 and **P < 0.01.

Transfection of PLNCaP and PC-3 cells with pcDNA-PLD5 overexpression (OE) or a combination of miR-145-5p mimics and PLD5 OE plasmid was undertaken to clarify miR-145-5p and PLD5 effects in prostate cancer. The cell proliferation investigation through MTT demonstrated a significant reduction of LNCaP (Figure 6a) and PC-3 cells (Figure 6b) following treatment with a combination of miR-145-5p mimics and PLD5 OE plasmid as compared to treatments with miR-145-5p mimics + pcDNA-NC, miR-145-5p mimics or NC. Cell invasion and migration assays also demonstrated a significant reduction following tumor cells treated with miR-145-5p + PLD5 OE in both LNCaP and PC-3 cells, as shown in Figure 6c, Figure 6d, and respectively. These data generally indicate the suppressive role of miR-145-5p in prostate cancer cell proliferation and metastasis.

## 4. Discussion

Prostate cancer (PCa) is reported to affect almost 21% of men, and death is predicted to affect 10% of the patients [20]. Even though various research has improved the PCa 5-year overall rate of survival to almost 100%, a significant drop to only 28% occurs in case of progression to a metastatic stage [21,22]. Gene therapy, such as microRNAs (miRNAs) is among the various approaches thought to prevent PCa metastasis, hence improving survival [23]. The current investigation assessed the role of miR-145-5p and PLD5 on PCa. The conclusions confirmed the inhibitory role of miR-145-5p and PLD5 on proliferation, invasion, and migration of PCa by targeting PLD5.

Several past studies have clarified the role of miR-145-5p as a suppressor of tumors on different kinds of malignancies. Pan et al. testified that miR-145-5p has a suppressive consequences on the invasion, migration, and proliferation of lung cancer cells by aiming the B-cell lymphoma 2 (Bcl-2), Bcl-2-associated X protein (Bax) and caspase-3 [Y. 24]. Further, downregulation of miR-145-5p has been reported on ovarian [25], bladder [26], and colon cancers [27]. Moreover, it was demonstrated that miR-145-5p is a tumor repressor, which has its expression regulated by P53 [X. 28]. Previous studies have shown miR145 downregulation in various tumors like B-cell, colorectal, ovarian, and mammary tumors [9].

Several miRNAs, which can serve prognostic roles, have been reported to be associated with PCa. A recent study presented that miR-206 binds with the androgen receptor and regulate the hormones in both breast and prostate cancer [29]. While miR-186-5p was found to play an oncogenic role in PCa. It was concluded that inhibition of miR-186-5p reduced PCa cell proliferation and invasion, and increased AKAP12 expression [30]. It was studied that targeting of MTDH by miR-145-5p or miR-145-3p is associated with PCa prognosis, thereby regulating the growth and metastasis of PCa cells [D. 13]. Meanwhile, it was also reported that miR-145-5p negatively regulates the level of SOX2 which exerts crucial role in PCa tumorigenesis and proliferation [2]. To further understand the role of miR-145-5p in PCa, the RT-qPCR findings of the current investigation affirmed its suppression. The expression of miR-145-5p has equally been shown to be repressed in other types of human cancers, for instance, gastric cancers [31]. The present study reported an inhibited migration, proliferation, and invasion as a consequence of miR-145-5p expression.

In assessing tumor repression mechanism, the present study hypothesized a regulation of PCa biological activities via corresponding genes’ modulation. Previous investigations have outlined genes such as MYO6 [31], ANGPT2 [K. 32], and TGF-β1 [Y. 33] as the ultimate target of miR-145. Several studies also investigated the role of PLD in tumor progression. Increased PLD biological activity has been associated with breast cancer progression [34]. Moreover, PLD1 overexpression promotes invasion and migration and function as a risk factor for Chinese glioma patients [35]. PLD1 has been linked with prostate cancer proliferation and colony formation [36]. However, the exact role of PLD5 has not been studied well, especially in cancer. Therefore, we aimed to study the linkage between PLD5 and miR-145-5p and ascertain PLD5 involvement in the progression of PCa. The RT-qPCR outcomes indicated a high PLD5 expression in PCa cells. To substantiate miR-145-5p and PLD5 interaction, dual-luciferase reporter and western blotting experiments confirmed the targeting of PLD-5 3ʹUTR by miR-145-5p, subsequently suppressing its expression. Our findings agreed with the hypothetical postulation to further elucidate on miR-145-5p tumor inhibition role via modulation of PLD5, whereby co-transfection with miR-145-5p mimics and PLD5OE upregulated proliferation, invasion, and migration of PCa.

Finally, assessment of the role of miR-145-5p mimicking on metastasis and proliferation through western blotting confirmed the suppression of N-cadherin, E-cadherin, Vimentin, and snail proteins, which indicated metastasis downregulation. The suppression of the AKT pathway also established the protective role of miR-145-5p on PCa proliferation. Similar to our study, some previous studies also reported that miRNAs regulate the expression of N-cadherin, E-cadherin, Vimentin, and snail proteins. For instance, miR-22 suppresses epithelial–mesenchymal transition in bladder cancer by inhibiting Snail and MAPK1/Slug/vimentin feedback loop [37]. Whereas miR-3622a expression inhibits EMT, progression and metastasis of PCa in vitro and in vivo by directly targeting EMT effectors ZEB1 and SNAI2 [38].

Summarily, this study gives a noble view on the link between PLD5 and miR-145-5p in PCa. Mimicking miR-145-5p suppresses PCa cells proliferation, invasion, and migration, miR-145-5p function via PD5 targeting in PCa, and the overexpression of miR-145-5p may rescue PLD5 oncogenic effects on PCa. In conclusion, the current research ascertained that miR-145-5p upregulated apoptosis and suppressed migration, invasion, and metastasis of PCa via direct PLD5 modulation. The work also offered in vitro and in vivo evidence to understand the effects of increased or decreased miR-145-5p or PLD5 in PCa tumor suppression or progression. Additionally, targeting of miR-145-5p/PLD5 could be an alternative therapeutic modality for PCa.

## Highlights

1. Relative expression of miR-145-5p is downregulated in PCa cell lines and tissues as compared to non-cancerous controls.
2. MiR-145-5p directly binds to the 3ʹ UTR of PLD5 which is a cancerous protein.
3. Overexpression of miR-145-5p diminishes the PLD5-oncogenic effects and inhibits PCa cells proliferation, migration, invasion, and metastasis.
